# The Ecological Response of *Carex lasiocarpa* Community in the Riparian Wetlands to the Environmental Gradient of Water Depth in Sanjiang Plain, Northeast China

**DOI:** 10.1155/2013/402067

**Published:** 2013-08-26

**Authors:** Zhaoqing Luan, Zhongxin Wang, Dandan Yan, Guihua Liu, Yingying Xu

**Affiliations:** ^1^Key Laboratory of Wetland Ecology and Environment Science, Northeast Institute of Geography and Agroecology, Chinese Academy of Sciences, 4888 Shengbei Road, Changchun 130102, China; ^2^University of Chinese Academy of Sciences, 19A Yuquan Road, Beijing 100049, China

## Abstract

The response of *Carex lasiocarpa* in riparian wetlands in Sanjiang Plain to the environmental gradient of water depth was analyzed by using the Gaussian Model based on the biomass and average height data, and the ecological water-depth amplitude of *Carex lasiocarpa* was derived. The results indicated that the optimum ecological water-depth amplitude of *Carex lasiocarpa* based on biomass was [13.45 cm, 29.78 cm], while the optimum ecological water-depth amplitude of *Carex lasiocarpa* based on average height was [2.31 cm, 40.11 cm]. The intersection of the ecological water-depth amplitudes based on biomass and height confirmed that the optimum ecological water-depth amplitude of *Carex lasiocarpa* was [13.45 cm, 29.78 cm] and the optimist growing water-depth of *Carex lasiocarpa* was 21.4 cm. The TWINSPAN, a polythetic and divisive classification tool, was used to classify the wetland ecological series into 6 associations. Result of TWINSPAN matrix classification reflected an obvious environmental gradient in these associations: water-depth gradient. The relation of biodiversity of *Carex lasiocarpa* community and water depth was determined by calculating the diversity index of each association.

## 1. Introduction

Water regime, as distinct from instantaneously measured water depth, has been implicated in affecting the composition, diversity, and distribution of macrophyte communities [1–6]. While the influence of water level fluctuation on the germination and establishment of wetland seed banks has been well documented, there is few research on the impact of water level on the growth of mature plants [2]. Water regime (depth, duration, and frequency of flooding) is the principal factor determining plant species distribution along the the land-water interface in wetlands [1, 6–17]. *Carex lasiocarpa* wetland is the main wetland type in the mire wetlands in Sanjiang Plain, Northeast China [18]. *Carex lasiocarpa*, a perennial Cyperaceae moss grass, is a clonal perennial which can form nearly monospecific stands on shorelines and lakesides [2, 19–23]. Where water conditions permit, such as in bays protected from waves, the species sometimes forms thick, floating mats. These floating mats often support a rich array of other plant life adapted to wet infertile conditions. Hence, this particular species of *Carex* is important in producing distinctive plant communities along lakes and rivers [2, 19, 20]. In wetlands in Sanjiang Plain it is generally considered to be an indicator species for wetlands [18, 19, 21, 22].

Responses of diversity of assemblages and individual species to water depth are necessary to be considered in the management and restoration efforts of wetland ecosystems; therefore, understanding plant response to hydrologic conditions is important both to the maintenance of native biodiversity and to the design of management strategies appropriate to a specific wetland [8, 24–26]. At present, research on the vegetation in Sanjiang plain wetlands still mostly focuses on the traditional classification and description, while little attention paid on the ecological pattern and the process of quantitative research, especially the quantitative research on the relationship between wetland hydrological and vegetation [2, 20, 21, 27–31]. The present study sought to achieve the following two objectives: (1) analyze the response of *Carex lasiocarpa* populations to the environmental gradient of water depth using the biological characters of biomass and height based on Gaussian Model and figure out the ecological amplitude of reed populations to water depth; (2) explore the relationship between the ecological characteristics of *Carex lasiocarpa *communities and water-depth variables. Based on the relationship between the ecological characteristics of wetland vegetation and hydrological regime, the suggestion of ecohydrological management for wetland ecosystem is proposed.

## 2. Materials and Methods

### 2.1. Study Area

**Figure 1:**
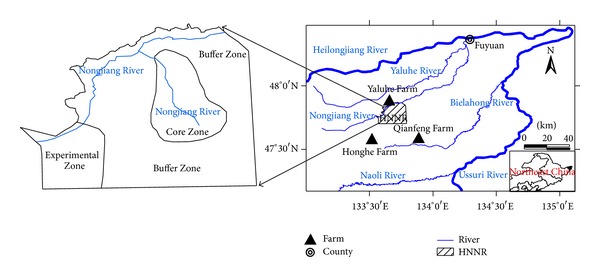
Location of the study area.

The Honghe National Nature Reserve (HNNR) (47°42′18′′N–47°52′N and 133°34′38′′E–133°46′29′′E) is located in the northeast of Sanjiang Plain, Northeast China, with an area of 250.9 ha (Figure 1).

HNNR has been listed as the International Important Wetland (Ramsar wetland) since 2001 for being a typical inland wetland and fresh water ecosystem in the north temperate zone [32]. Presently, HNNR has 16 orders, 43 families, and 174 species of waterfowl, including ten species of nationally rare and endangered waterfowl. In addition, 1012 species of plants are founded in HNNR, including six species of nationally endangered plants. With a very low topographic gradient (average slope grade less than 1 : 10,000), this area is favorable to the formation of wetland ecosystems [33].

### 2.2. Methods

#### 2.2.1. Sampling Method

**Figure 2:**
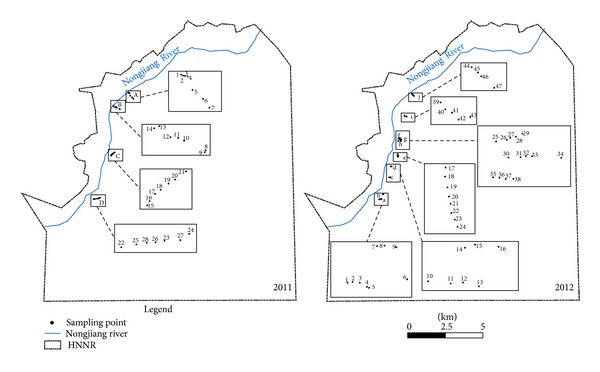
Location of sampling spots.

Samples were collected in 28 sampling spots (Figure 2) from May to September of 2011 and 47 sampling spots in 2012. Three quadrats (50 × 50 cm^2^) of plants samples were collected with scissors at each sample point. Plants naturally growing in the quadrats were recorded with their names, abundances, coverage, heights, and aboveground biomass (dry weight). Coordinate of each sampling quadrat was also recorded by using GPS.

#### 2.2.2. Data Analysis

(1) Gaussian Model was adopted to describe the species-environmental relations. The study on the response of reed to water depth based on the Gaussian Model has been achieved good effect [34, 35]. The Gaussian Model was shown as the following equation:

(1) $$\begin{array}{c} y=ce^{\left[-(1/2){\left(x-u\right)}^{2}/t^{2}\right]}, \end{array}$$

where *y* represents an indicator of biological characteristics of plant species, which can be abundance, coverage, density or biomass, and so on; *c* is the maximum of *y*; *x* is the value of environmental factor, *u* is the optimum ecological amplitude of species to environmental factor; and *t* is species tolerance. Generally optimum ecological amplitude of species to environmental factor change within 2*t* range. The analysis was performed by Excel 2003.

(2) Two-Way Indicator Species Analysis (TWINSPAN) was used to classify the plant community in the study area based on the number of species in all quadrats [36]. The TWINSPAN analysis was performed by using winTWINS 2.3 [37].

(3) The ecological characteristics of plant community were reflected with the following indices [38–40].

Species richness: Margalef index (MA) was adopted, which is expressed as

(2) $$\begin{array}{c} \text{MA}=\frac{\left(S-1\right)}{\ln N}, \end{array}$$

where *S* is the number of species and *N* is the number of individuals of all species in a community.

Species diversity: Shannon-Weaver index (*H*) was used, which can be calculated as

(3) $$\begin{array}{c} H\;=-\Sigma Pi\ln Pi, \end{array}$$

where *Pi* = *ni*/*N*, *ni* is the importance value of species *i*, and *N* is the sum of the importance value of all species in a community.

Species evenness: Pielou evenness index (*E*) was used, which is expressed as:

(4) $$\begin{array}{c} E=\frac{H}{\ln\left(S\right)}, \end{array}$$

where *S* is the number of species and *H* is the Shannon-Weaver index.

(4) Data was analyzed by using SPSS17.0 and Microsoft Excel.

## 3. Results and Discussion

### 3.1. Response of *Carex lasiocarpa* to Water Depth

#### 3.1.1. Result of Statistical Analyses

17* Carex lasiocarpa* community sampling data (spots without *Carex lasiocarpa* removed) from May to September in 2011 were statistically analyzed (Table 1). The water depth ranged within 2.5–37.5 cm with a mean value of 17.18 cm. Average population height ranged within 38–73.25 cm, and the average value was 59.64 cm. Range of populations biomass was 1.3–47.68, and the mean value was 25.37. All the sampling data was of normal distribution.

#### 3.1.2. Response of the Population Biomass of* Carex lasiocarpa* to Water Depth

**Figure 3:**
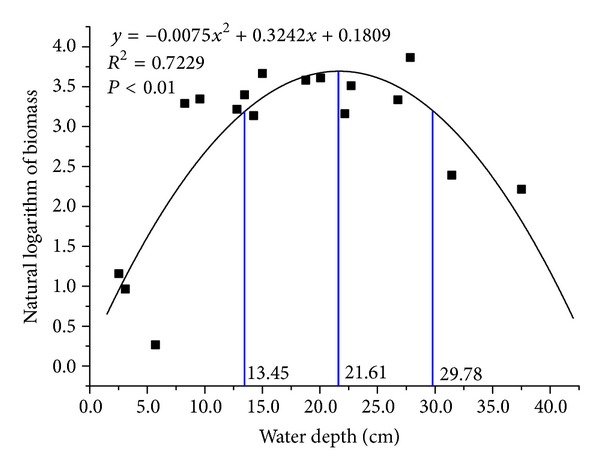
The secondary nonlinear regression based on Gaussian Model of *Carex lasiocarpa* population biomass and water depth.

Population biomass of* Carex lasiocarpa* was strongly correlated to water depth (*R* ^2^ = 0.7229, *P* < 0.01). Quadratic curve fitting was used to fit the relationship between population biomass data of *Carex lasiocarpa *(after natural logarithm transformation) and water-depth data, and the obtained quadratic curve was fit with gaussian regression (Figure 3). With regression analyses, the Gaussian regression equation and regression curve were obtained, which was expressed as the following equation:

(5) $$\begin{array}{c} y=39.82\exp\left[-\frac{\left(1/2\right){\left(x-21.61\right)}^{2}}{8.16^{2}}\right]. \end{array}$$

The results indicated that the optimum ecological amplitude of *Carex lasiocarpa* to water depth based on population biomass was [13.45 cm, 29.78 cm] and the optimist growing point is 21.6 cm.

#### 3.1.3. Response of the Population Height of *Carex lasiocarpa* to Water Depth

**Figure 4:**
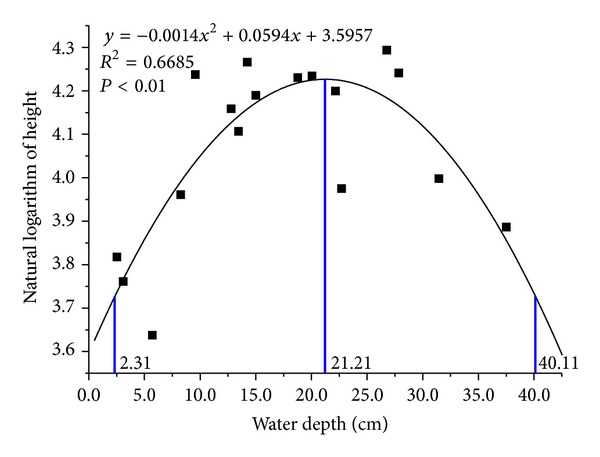
The secondary nonlinear regression based on Gaussian Mode of *Carex lasiocarpa* populations height and water depth.

Population height of *Carex lasiocarpa* was strongly correlated to water depth (*R* ^2^ = 0.6685, *P* < 0.01). Quadratic curve fitting was used to fit the relationship between population height data of *Carex lasiocarpa* (after natural logarithm transformation) and water-depth data, and the obtained quadratic curve was fit with Gaussian regression (Figure 4). With regression analysis, the Gaussian regression equation and regression curve were obtained, which was expressed as the following equation:

(6) $$\begin{array}{c} y=68.43\text{ exp}\left[-\frac{\left(1/2\right){\left(x-21.21\right)}^{2}}{18.90^{2}}\right].\; \end{array}$$

The results indicated that the optimum ecological amplitude of *Carex lasiocarpa *to water depth based on population average height was [2.31 cm, 40.11 cm] and the optimist growing point is 21.2 cm.

#### 3.1.4. The Optimum Ecological Amplitude of *Carex lasiocarpa* to Water Depth

An intersection of the ecological amplitudes based on biomass ([13.45 cm, 29.78 cm]) and height ([2.31 cm, 40.11 cm]) was carried out to figure out the ecological amplitude of *Carex lasiocarpa* to water depth in general. The final result confirmed that the optimum ecological amplitude of *Carex lasiocarpa* to water depth was [13.45 cm, 29.78 cm] and the optimist growing point of *Carex lasiocarpa* to water depth was 21.4 cm.

### 3.2. Response of Community Diversity of *Carex lasiocarpa* to Water Depth

**Figure 5:**
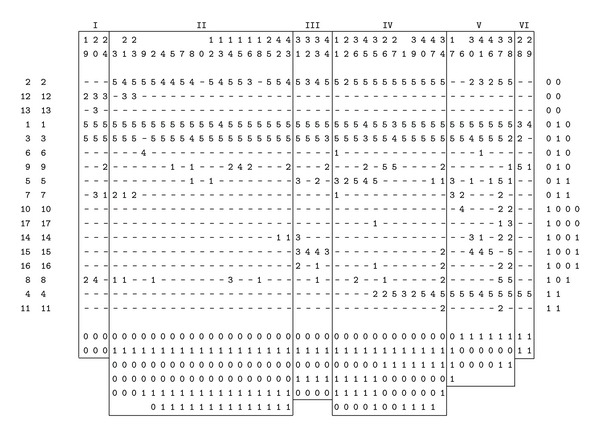
TWINSPAN analyses. Note: *1-Carex lasiocarpa, 2-Glyceria spiculosa, 3-Carex pseudo-curaica, 4-Calamagrostis angustifolia, 5-Galium manshuricum Kitag., 6-Galium dahuricum Turcz, 7-Comarum palustre L., 8-Equisetum fluviatile, 9-Carex humida, 10-Phragmites australis, 11-Anemone dichotoma, L. 12-Menyanthes trifoliate, 13-Achillea acuminate, 14-Lathyrus quinquenervius., 15-Carex dispalata, 16-Salix rosmarinifolia, L. 17-Caltha palustris var, sibirica.*

By using TWINSPAN, the 47 sampling spots in 2012 were classified into 6 groups at the end of division (Figure 5).

Association Group I: Association *Carex pseudocuraica + Carex lasiocarpa*. This was also a hygrophyte association group including 3 sampling spots (S19, S20, S24).* Carex pseudo-curaica* and *Carex lasiocarpa* were dominant species, while the others were companion species. *Carex pseudo-curaica* occurred in relatively deep water conditions.

Association Group II: Assoc.* Carex pseudo-curaica + Carex lasiocarpa + Glyceria spiculosa*. This was also a hygrophyte association group including 19 sampling spots (S2, S3, S4, S5, S7, S8, S9, S10, S12, S13, S14, S15, S16, S18, S21, S23, S25, S42, S43). The group occured in relatively moderate water-depth conditions.

Association Group III: Assoc. *Carex lasiocarpa + Carex pseudo-curaica + Glyceria spiculosa + Carex dispalata.* This was also a hygrophyte association group including 4 sampling spots (S31, S32, S33, S44). The group occured relatively in moderate water depth conditions.

Association Group IV: Assoc. *Glyceria spiculosa + Carex lasiocarpa + Carex pseudo-curaica + Calamagrostis angustifolia*. This was also a mesophyte association group including 12 sampling spots (S1, S11, S22, S26, S27, S34, S35, S36, S39, S40, S45, S47).

Association Group V: Assoc. *Carex lasiocarpa* + *Calamagrostis angustifolia* + *Carex pseudo-curaica*. This was also a mesophyte association group including 7 sampling spots (S6, S17, S30, S37, S38, S41, S46). *Carex lasiocarpa*, *Calamagrostis angustifolia* and *Carexpseudo-curaica* were dominant species, and the others were companion species.

Association Group VI: Assoc.* Calamagrostis angustifolia + Carex lasiocarpa*. This was also a mesophyte association group including 2 sampling spots (S28, S29). *Calamagrostis angustifolia* occurred in relatively shallow water conditions.

TWINSPAN classification matrix results reflected an obvious environmental gradient: water depth. The weighted-average wetland indicator status for each community type reflected the distribution of community types along the hydrologic gradient. The matrix diagram reflected that from association 1 to association 6 the water depth was gradually reduced, which determined the distribution range of these species.

Based on the inquisitional data of sampling sites, the biodiversity of the *Carex lasiocarpa* community in HNNR was analyzed by adopting diversity index, richness index, and evenness index.

**Figure 6:**
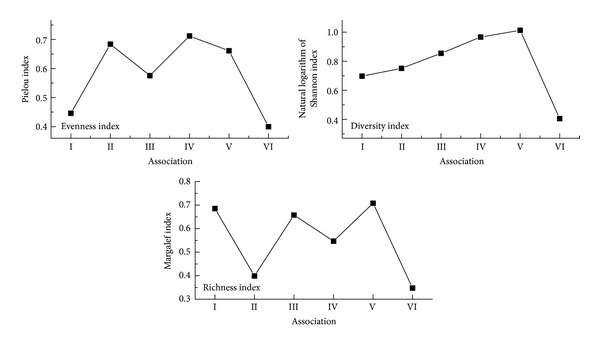
*Carex lasiocarpa* community biodiversity index.

The characteristics of the plant community can reflect vegetation functions and ecological niche. In our research, the characteristics of plant community (species richness MA, species diversity index *H*, and species evenness index *E*) were supposed to be different among different water-depth areas. Therefore, 47 quadrats were divided into 5 groups based on different water depth, and then the biodiversities indices of plant community were analyzed (Figure 6). The results demonstrated that the water depth was distinct in different vegetation types, and the optimal water depth for *Carexpseudo-curaica* was the deepest, followed by* Glyceria spiculosa*,* Carex lasiocarpa*, and *Calamagrostis angustifolia*. It was observed that the evenness index and diversity index of* Carex lasiocarpa* communities were low when water depth was too high or too low, while species evenness was poorer. When the water depth was moderate, *Carex lasiocarpa* species distributes evenly (Figure 5). Species richness of association I was high because of relatively more species, but when water depth was too high, *Carex pseudocuraica* and *Carex lasiocarpa* occured as dominant species, and other associated species were rare. *Carex pseudocuraica*, *Carex lasiocarpa,* and *Glyceria spiculosa* in association II were main dominant species, accompanying species was less, therefore species richness was low. The number of species in association III, IV, and V was more, and evenness and abundance of plant communities were higher. The growth of *Carex lasiocarpa* community vegetation was significantly correlated with water depth, which can be found from biodiversity of each association. With the increase or decrease of water depth, density of *Carex lasiocarpa* community decreased while biodiversity increased.

## 4. Conclusions

The results indicated that the optimum ecological amplitude of *Carex lasiocarpa* to water depth based on population biomass was [13.45 cm, 29.78 cm], while the optimum ecological amplitude of *Carex lasiocarpa* to water depth based on average height was [2.31 cm, 40.11 cm]. The optimum ecological amplitude of *Carex lasiocarpa* to water depth was [13.45 cm, 29.78 cm] and the optimist growing point of *Carex lasiocarpa* to water depth was 21.4 cm.

TWINSPAN classification matrix results reflected an obvious environmental gradient for wetland plant species: water-depth gradient. *Carex lasiocarpa* maintains high cover across most water-depth gradients but requires high variation at the wettest conditions. Water depth for plant species in the freshwater marsh showed the order as *Carex pseudocuraica* > *Carex lasiocarpa* > *Glyceria spiculosa* > *Calamagrostis angustifolia.*

The growth of *Carex lasiocarpa* community was significantly correlated with water depth. With the increase or decrease of water depth, the densityof *Carex lasiocarpa* decreased, and the evenness index and diversity index of* Carex lasiocarpa* communities were low. In moderate water depth condition, the density of *Carex lasiocarpa* was the highest.

## Figures and Tables

**Table 1:** The statistical description of *Carex lasiocarpa* community in different sampling points.

Sampling points	Water depth (cm)	Population height (cm)	Population biomass (g/m^2^)
2	22.70	53.25	33.54
4	12.80	64.00	24.96
5	8.20	52.50	26.86
6	5.70	38.00	1.30
7	3.10	43.00	2.63
8	26.80	73.25	28.10
9	27.90	69.50	47.68
10	20.10	69.00	36.90
12	18.80	68.75	35.92
13	13.50	60.75	29.94
17	31.50	54.50	10.92
19	37.50	48.75	9.15
21	22.20	66.67	23.58
23	9.60	69.25	28.41
25	15.00	66.00	39.00
26	14.20	71.25	46.05
28	2.50	45.50	6.36
Mean	17.18	59.64	25.37
Max	37.50	73.25	47.68
Min	2.50	38.00	1.30
